# Mannoprotein MP84 mediates the adhesion of *Cryptococcus neoformans* to epithelial lung cells

**DOI:** 10.3389/fcimb.2014.00106

**Published:** 2014-08-19

**Authors:** Pedro A. C. Teixeira, Luciana L. Penha, Lucia Mendonça-Previato, Jose O. Previato

**Affiliations:** Laboratório de Glicobiologia, Instituto de Biofísica Carlos Chagas Filho, Universidade Federal do Rio de JaneiroRio de Janeiro, Brazil

**Keywords:** *Cryptococcus neoformans*, mannoprotein MP84, cell adhesion, A549 epithelial lung cell

## Abstract

The capsule is the most important virulence factor of the fungal pathogen *Cryptococcus neoformans*. This structure consists of highly hydrated polysaccharides, including glucuronoxylomannan (GXM), and galactoxylomannan (GalXM). It is also composed of mannoproteins (MPs) which corresponds to less than 1% of the capsular weight. Despite MPs being the minority and least studied components, four of these molecules with molecular masses of 115, 98, 88, and 84 kDa were identified and characterized as *C. neoformans* immunoreactive antigens involved in the pathogenesis, and are potential cryptococcosis vaccine candidates. With the aim to describe the adhesive property of MPs, we cloned and expressed the MP84, a mannoprotein with molecular weight of 84 kDa, on *Pichia pastoris* yeast, and performed interaction assays of *C. neoformans* with epithelial lung cells, in the presence or absence of capsule components. Two fungal strains, the wild type, NE-241, and a mutant, CAP67, deficient in GXM production, were used throughout this study. The adhesion assays were completed using epithelial lung cells, A549, and human prostate cancer cells, PC3, as a control. We observed that capsulated wild type (NE-241), and acapsular (CAP67) strains adhered significantly to A549 cells, compared with PC3 cells (*p* < 0.05). GXM inhibits the NE-241 adhesion, but not the CAP67. In contrast, CAP67 adhesion was only inhibited in the presence of MP84. These results demonstrate the involvement of MP in the adhesion of *C. neoformans* to epithelial lung cells. We conclude that this interaction possibly involves an adhesion-like interaction between MP on the fungal surface and the complementary receptor molecules on the epithelial cells.

## Introduction

The opportunistic fungus *Cryptococcus neoformans* is the etiological agent of cryptococcosis, a disease that kills about 630,000 people per year globally (Park et al., 2009). This infection is most probably acquired by inhalation of desiccated cells, which are present in the environment as basidiospores or poorly encapsulated yeasts (Rodrigues et al., 1999). This disease can manifest into different clinical forms, with the most severe being cryptococcal meningitis that affects mainly immunocompromised patients, such as individuals with HIV/AIDS. Although the incidence of HIV-associated cryptococcosis has decreased in developed countries since the introduction of antiretroviral therapy against AIDS, this disease continues to cause significant morbidity and mortality in the less developed world (La Hoz and Pappas, 2013).

*C. neoformans* has certain attributes that provide their survival in specific ecological niches. Some attributes include the ability to grow at 37°C and melanin production. These are indispensable for the adaptation into the mammalian host environment. However, the cryptococcal capsule, which surrounds the cell body, is considered the major determinant of virulence of this pathogen, with potent anti-phagocytic properties (Zaragoza et al., 2009). Many microbes possess capsules that play important roles, for example, in resistance to stressful conditions (such as dehydration), and in the interaction with the environment (Zaragoza et al., 2009). The *C. neoformans* capsular network consists of a highly hydrated polysaccharide gel, composed of high-molecular weight polysaccharide polymers, such as glucuronoxylomannan (GXM), which represents almost 90% of the total capsule; the remainder being galactoxylomanna (GalXM) (Kumar et al., 2011). GXM is composed of a large backbone of 6-O-acetylated α-1,3-mannose residues with β-D-xylopyranosyl, β-D-glucuronosyl monosubstituted side chains (Cherniak and Sundstrom, 1994). Recently, Heiss et al. (2009) re-examined the structure of *C. neoformans* GalXM by Nuclear Magnetic Resonance (NMR) spectroscopy and Gas-liquid Chromatography-Mass Spectrometry (GC–MS), and proposed GalXM to be termed glucuronoxylomannogalactan (GXMGal).

The complexity of the cell surface architecture of *C. neoformans* is increased by the presence of mannoproteins (MPs), that comprises less than 1% of the capsule mass (Rodrigues and Nimrichter, 2012). These proteins are highly mannosylated antigens, usually containing 80–90% of mannose, and found in a wide range of fungal species (Zhang and Ballou, 1981; Murphy, 1988; Cao et al., 1998; Nguyen et al., 1998; Nisini et al., 2001; Frieman et al., 2002; Mansour and Levitz, 2003; Chong et al., 2004). The *C. neoformans* MPs were first identified in culture filtrates (Reiss et al., 1985; Murphy, 1988) and were defined as MPs by their ability to adhere to a Concanavalin A (Con A) affinity column (Murphy et al., 1988).

Investigation on the location of MPs on cryptococcal cells concluded that they are mainly found in the inner capsule, near the cell wall, and not associated with GXM or GXMGal (Vartivarian et al., 1989; Jesus et al., 2010).

The role of MPs in capsule architecture has never been established. However, their effects on the host cells have long been studied. It is well known that *C. neoformans* MPs are highly immunogenic. Some authors consider MPs as a key inflammatory mediator that induces a protective immune response against *C. neoformans* infection, promoting them as a vaccine candidate against fungi (Pietrella et al., 2001, 2005; Levitz and Specht, 2006).

Several *C. neoformans* MPs have been isolated and new MP roles have been identified, besides the immunogenic function. In 2002, a specific MP involved in T-cell activation was identified. This protein had an apparent molecular weight of 88 kDa, and was therefore named MP88 (Huang et al., 2002). One year earlier, a 98 kDa MP involved in the stimulation of T-cell responses was identified and named MP98. Analysis of the predicted amino acid sequence of MP98 reveals a domain with chitin deacetylase activity (Levitz et al., 2001). In 2005, new immunogenic MPs have been identified, with molecular weights of 250, 125, 115, and 84 kDa. The genes encoding MP115 and MP84 were cloned and had homology with carboxylesterases and chitin deacetylases, respectively (Biondo et al., 2005). Recently, the MP Cig1 was described to be a mediator of iron uptake, functioning as a hemophore at the cell surface, and contributing to virulence in a mouse model of cryptococcosis (Cadieux et al., 2013).

Inhalation of *C. neoformans* is the main pathway for infection. An effective interaction of this fungus with epithelial alveolar cells is crucial for disease establishment. Once adhered to the pulmonary epithelia, *C. neoformans* can proliferate and induce primary lesions in the lung (Kawakami, 2004). A previous study suggested that *C. neoformans* can use GXM for attachment to epithelial lung cells (Barbosa et al., 2006). Since the alveolar epithelium is the first host site to be challenged by *C. neoformans* to establish a successful infection, we aimed to clone and express the MP84 on *Pichia pastoris* yeast, and investigate the role of this MP in the interaction of *C. neoformans* with epithelial lung cells. We found that these cells apparently express MPs binding sites responsible for the attachment of *C. neoformans* to A549 monolayers.

## Materials and methods

### Microorganism and culture conditions

Two *C. neoformans* strains were used throughout this study, a wild type strain, NE-241, and a mutant strain, deficient in GXM production, CAP67. They were kindly provided by Professor Tamara Doering (Department of Molecular Microbiology, Washington University School of Medicine, St Louis, MO, USA) and Professor Robert Cherniak (Georgia State University, Atlanta, GA, USA), respectively. The strains were grown on YPD agar, for 72 h at 37°C and stored at 4°C as stock cultures. To obtain free cells, NE-241 and CAP67 were cultivated at 37°C under constant shaking (150 rpm) for 120 h, in a chemically defined CDCB 2550 medium containing (g/L) dextrose, 20; urea, 1.29; KH_2_PO_4_.H_2_O, 1.36; MgSO_4_.7H_2_O, 0.3; sodium glutamate, 1.0; thiamine-HCl, 2 × 10^−3^; biotin, 1 × 10^−5^. Yeast cells were obtained by centrifugation and washed twice in 0.01 M phosphate buffered saline (PBS), pH 7.2. Cell growth was estimated by counting the number of yeasts in a Neubauer chamber. Capsule expression was determined by India ink staining.

### A549 epithelial lung cells

A549 is a human type II alveolar epithelial-like lineage, which is derived from lung carcinomatous tissue of a human patient, and is widely used as a model for infection of respiratory pathogens (Hahn, 1997). The cultures were maintained and grown to confluence in 25 cm^2^ culture flasks containing Dulbecco's modified Eagle's medium (DMEM) supplemented with 10% fetal bovine serum (FBS), at 37°C in a 5% CO_2_ atmosphere. For interaction experiments with *C. neoformans* or ELISA with recombinant MP84, A549 cells were cultivated on 96 or 24-well plates, respectively.

### Isolation and purification of capsular polysaccharides

The capsular components GXM, GXMGal and MPs were obtained from the NE-241 strain, which was grown in a chemically defined medium (CDCB 2550). These capsular polysaccharides were isolated and purified as previously described by our group (Villena et al., 2008). Briefly, the culture supernatant was separated from cells by centrifugation (6000 *g*, for 30 min at 4°C) and precipitated by addition of three volumes of cold ethanol. The precipitate was collected by centrifugation, dissolved and dialyzed against distilled water and lyophilized. The lyophilized material was solubilized in distilled water and precipitated with cetyltrimethylammonium bromide (CTAB). The mixture was maintained for 18 h at room temperature, for precipitation. The precipitated material was suspended in 10% ethanol, centrifuged and the pellet was solubilized in 1M NaCl. This solution was precipitated with three volumes of ethanol; the pellet was solubilized in distilled water, dialyzed and lyophilized, resulting in the GXM fraction. The supernatant was concentrated and precipitated in three volumes of ethanol. The pellet was solubilized in distilled water, dialyzed and lyophilized, resulting in the GXMGal+MP fraction.

### *C. neoformans* RNA extraction and cDNA obtainment

*C. neoformans* RNA was extracted from CAP67 strain using Brazol reagent (LGC Biotecnologia). Briefly, 0.75 ml of Brazil was added to 5 × 10^6^ CAP67 yeast cells and incubated under room temperature for 5 min; 0.2 mL of chloroform was then added and incubated at room temperature for 3 min. The suspension was centrifuged (12,000 *g*) for 15 min at 4°C, and 0.5 ml of 100% isopropanol was added to the aqueous phase. After incubation at room temperature for 10 min, the tube was centrifuged (12,000 *g*) for 10 min at 4°C. The pellet was washed with 75% ethanol and resuspended in *Rnase free* MilliQ water. Any DNA genomic contamination was removed by treatment of 5 μg of the total RNA with 1 U RQ1 RNase-free DNas I (Promega) for 15 min at 37°C.

The cDNA was obtained from the extracted RNA (1 μg), using the RevertAid H Minus First Strand cDNA Synthesis Kit (Fermentas Life Science), as described in the manufacturer's manual.

### MP84 gene amplification and cloning

The gene encoding the 84 KDa MP was amplified by Polymerase Chain Reaction (PCR), using *Taq* DNA polymerase (Promega) with oligonucleotides 5′ATA*CTGCAG*GGCACGAGTCATGGCTTCAGCC 3′ (sense) and 5′ATA*TCTAGA*CCACTACCGCGTGGCACCAGTTGGGAGCTGGCAGCAGAGG 3′ (antisense) (the underlined regions are restriction sites of *Pst*I and *Xba*I enzymes, respectively). This consisted of an initial denaturation step of 5 min at 95°C; 25 cycles of 45 s at 95°C, 45 s at 55°C, and 60 s at 72°C; and a final extension step for 7 min at 72°C. The amplified fragments were purified from agarose gels using Gel Extraction Kit (Qiagen).

To clone the MP84 gene, pPICZαB plasmid (Invitrogen) and purified amplified fragment were digested with *Pst*I and *Xba*I restriction enzymes (New England Biolabs), and the ligation was performed with T_4_ DNA ligase enzyme (Promega). After transformation in *Escherichia coli* DH5α, some clones were selected to performance the Mini-prep using Gel Extraction Kit (Qiagen) and differential digestion assays were done to confirm the cloning. The positive clones were sequenced in an API-3100 (Applied Biosystems) automated sequencer and sequences were analyzed by DNASTAR Lasergene software (Version 7.2).

### *Pichia pastoris* transformation

*P. pastoris* X-33 strain was used to express and secrete recombinant MP84 in culture medium. After sequence analysis, the pPICZαB plasmid with MP84 gene was digested by *Bgl*II restriction enzyme (New England Biolabs), for 1 h at 37°C, to linearize the plasmid. The transformation occurred by the addition of 5 μL of linearized plasmid (10 μg/μL) to the competent *P. pastoris* cells, suspended in 1 M cold Sorbitol, and incubated for 5 min on ice before electroporation. The electroporator (BioRad) was set at 2.43 kV, 25 μF, and 400 Ω, under pulse time of 9.6. An aliquot of 600 μL 1 M cold Sorbitol was added to the electroporated cell suspension, followed by incubation at 30°C for 30 min. YPD medium was added to the cell suspension and incubated for an additional 30 min at 30°C. The cells were then plated on YPD agar with 100 μg/mL zeocin (Invitrogen) and cultivated at 28°C for 48 h to select the transformants.

### Expression test

With the aim to select the colony that expressed and secreted recombinant MP84 the most, 5 colonies were randomly chosen and cultivated overnight at 28°C on BMGY (buffered complex glycerol medium). After centrifugation, the cells were transferred to BMMY (buffered complex methanol medium) for induction of recombinant MP84 expression. 100% methanol was added for a final concentration of 0.5% for each colony at 24 h of growth. Culture medium samples were collected on each colony at 24, 36, 48, 60, 72, 84, and 96 h. These samples were analyzed by Western Blot, as described in detail elsewhere (Lam et al., 2005), using anti-histidine antibody (Pierce) and mouse anti-IgG HRP conjugated and revealed with SuperSignal West Pico Chemiluminescent Substrate (Pierce), to determinate recombinant MP84 expression levels and the duration of the secretion of this protein for each *P. pastoris* colony. A colony with pPICZαB without MP84 gene was used as negative control.

### Purification of recombinant MP84

Recombinant MP84 was purified by an immobilized metal affinity chromatography (IMAC) using precharged Ni Sepharose™ High Performance (His Trap HP, Amersham Biosciences), as described by manufacturer's manual. The selected *P. pastoris* colony was grown on 2 L of BMMY medium with addition of 0.5% methanol every day at 28°C, for the time selected. After centrifugation, the culture supernatant was applied in a nickel affinity column (His Trap HP column), which recognizes the histidine tag added to the recombinant MP84 during molecular biology steps. Elution buffer (20 mM sodium phosphate, 0.5 M NaCl, 500 mM imidazole, pH 7.4) was used to elute the recombinant MP84. This protein was then dialyzed against 20 mM sodium phosphate, 0.5 M NaCl to remove imidazole. Before storing at −80°C, the eluate was analyzed by SDS-PAGE, and stained with comassie blue to verify the purity of the sample.

### Interaction of *C. neoformans* with epithelial lung cells

A549 epithelial lung cells and PC3 prostate human epithelial cells were cultivated on 24-well plates on DMEM supplemented with 10% fetal bovine serum (FBS), at 37°C in a 5% CO_2_ atmosphere, for about 36 h until the monolayer formation. Each well was then inoculated with 10^6^ *C. neoformans* yeast cells suspended on DMEM medium to a final volume of 500 μL per well, and incubated at 37°C for 1 h. For inhibition assays, A549 cells were pre-incubated with GXM, GXMGal+MP or MP84, at 37°C for 1 h. After three washings with DMEM medium to remove non-adhered cells, the A549 cells were lysed with sterile cold water. The cell lysate was recovered and plated onto YPD agar and incubated at 37°C. After 48 h, the number of colony-forming units (CFU) was determined. The experiment was performed in triplicate and statistically analyzed by using Student's *t*-test. *p* < 0.05 was considered statistically significant.

### Binding of MP84 to epithelial lung cells

The binding between recombinant MP84 and A549 cells was evaluated by ELISA assays. A549 cells were suspended in DMEM and placed into the wells of a 96-well plate and cultivated at 37°C until the cells were confluent. Then, the lung cells were incubated with recombinant MP84 in DMEM, for 1 h at 37°C. After three washings with PBS containing 0.05% Tween 20 (PBS-T), the A549 cells were fixed with 4% of paraformoldehyde and blocked with 0.5% casein in PBS-T for 1 h at 37°C. After washing with PBS-T, cells were incubated with 1:3000 anti-histidine antibody (GE Healthcare) in PBS-T for 1 h at room temperature, washed, and incubated with mouse anti-IgG HRP conjugated (Cell Signaling) diluted 1:2000 under the same conditions. After washing, the reaction was revealed by TMB substrate solution (eBioscience). The absorbance was determined on the ELISA reader (Beckman Counter) at 450 nm. Negative control was performed by replacing the recombinant MP84 with PBS. The experiment was performed in triplicate and statistically analyzed by using Student's *t*-test. *p* < 0.05 was considered statistically significant.

## Results

### *C. neoformans* RNA extraction and MP84 gene amplification

**Figure 1:**
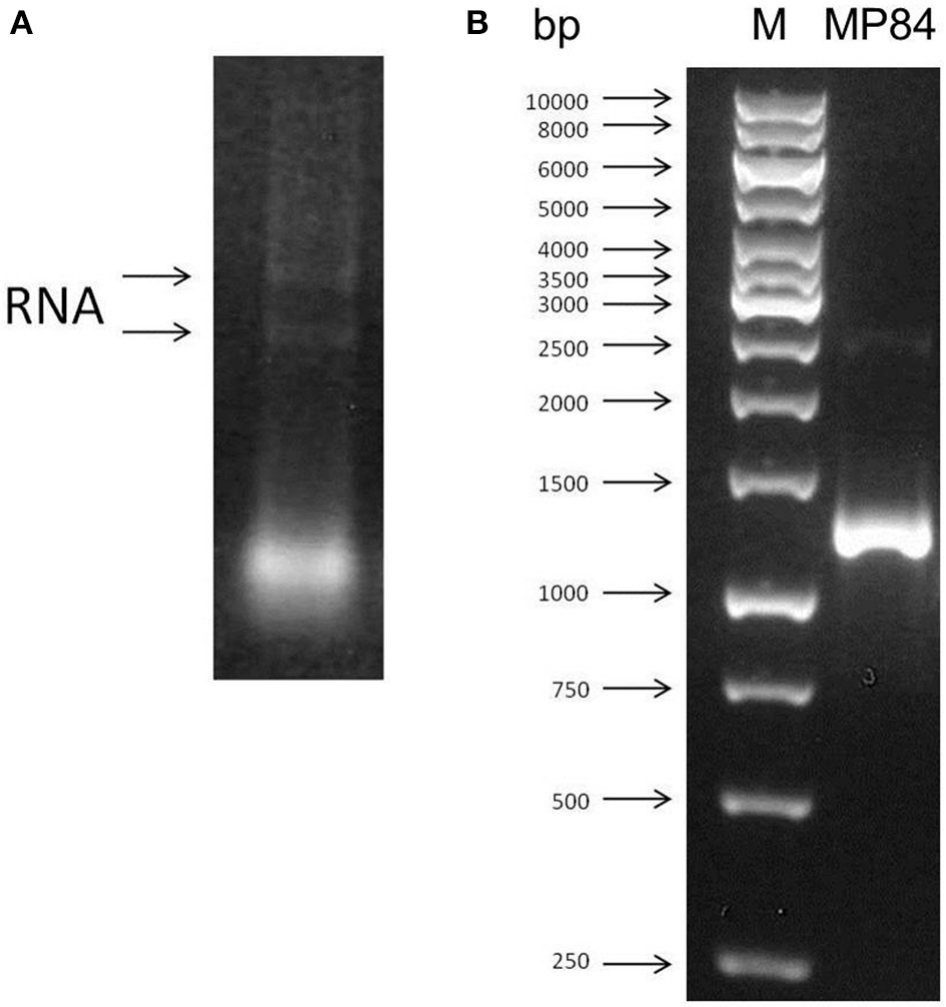
**RNA extraction and amplification of MP84 gene. (A)** Ethidium bromide-stained 1% agarose gel of total RNA (0.5 μg) extracted from *C*. *neoformans* CAP67. **(B)** PCR fragment amplified using specific oligonucletides for MP84. (M) Markers (BioTools).

With the aim to extract the *C. neoformans* total RNA, the poorly capsulated strain, CAP67, was grown for 18 h. The RNA was extracted with Brazol reagent as described in the Materials and Methods. The integrity and DNA free appearance were observed by agarose gel electrophoresis (Figure 1A). The extracted RNA was quantified and used to synthesize the *C. neoformans* cDNA, which was utilized as a template for the obtainment of the MP84 gene. The amplification of this gene was performed by PCR using high fidelity enzymes. Oligonucleotides driven to the coding region of MP84 resulted in the amplification of a single fragment of 1230 bp (Figure 1B).

### Cloning MP84 in pPICZαB plasmid

Competent *E. coli* DH5α bacteria were transformed with insert-vector, and were grown in culture medium containing zeocin antibiotic to screen for transformants. Some colonies had their plasmid extracted, linearized and analyzed on an electrophoresis gel to select the positive clones (data not shown).

**Figure 2:**
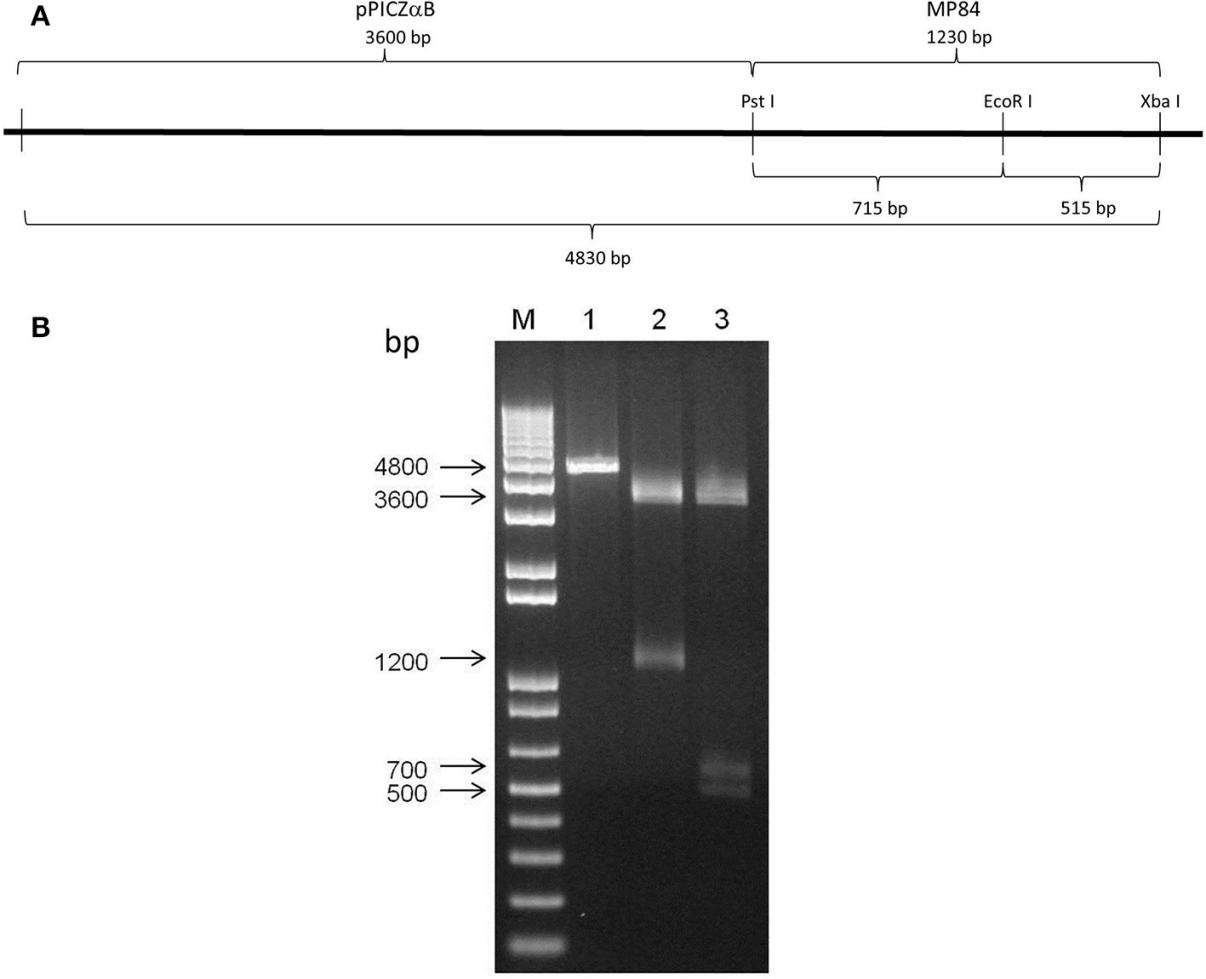
**Cloning of MP84 mannoprotein in pPICZαB plasmid**. The amplified gene of MP84 by PCR was ligated in expression vector pPICZαB and then transformed into *E. coli* DH5α. After the selection of the positive clones, the plasmids were extracted and the differential digestion was performed. All positives clones were sequenced to confirm the correct sequence. **(A)** Schematic representation of clone pPICZαB-MP84 and the restriction enzymes map. **(B)** pPICZαB-MP84 (lane 1) digested with restriction enzymes *Pst*I and *Xba*I (lane 2) and with *Pst*I, *Eco*RI and *Xba*I (lane 3).

To confirm the positive clones of MP84 gene in pPICZαB plasmid, the DNA of some clones was digested with *Pst*I and *Xba*I restriction enzymes, which produced two fragments of approximately 3600 and 1200 bp of molecular weight, corresponding to the correct size of pPICZαB and MP84, respectively (Figure 2B, lane 2). When the same insert-vector was digested with *Pst*I, *Eco*RI, and *Xba*I restriction enzymes, we observed three fragments with at approximately 3600, 700, and 500 bp, corresponding to the vector and two insert parts (Figure 2B, lane 3). The presence of fragments with molecular weight corresponding to the expression vector, the insert and the insert cut, suggests that the insertion of the MP84 gene in the pPICZαB plasmid was successful.

The sequences analyzed in DNASTAR software showed that the insert without mutation was in frame with the α-factor signal sequence as well as with the C-terminal c-myc and HIS_6_ (data not shown).

### *P. pastoris* transformation and purification of recombinant MP84

**Figure 3:**
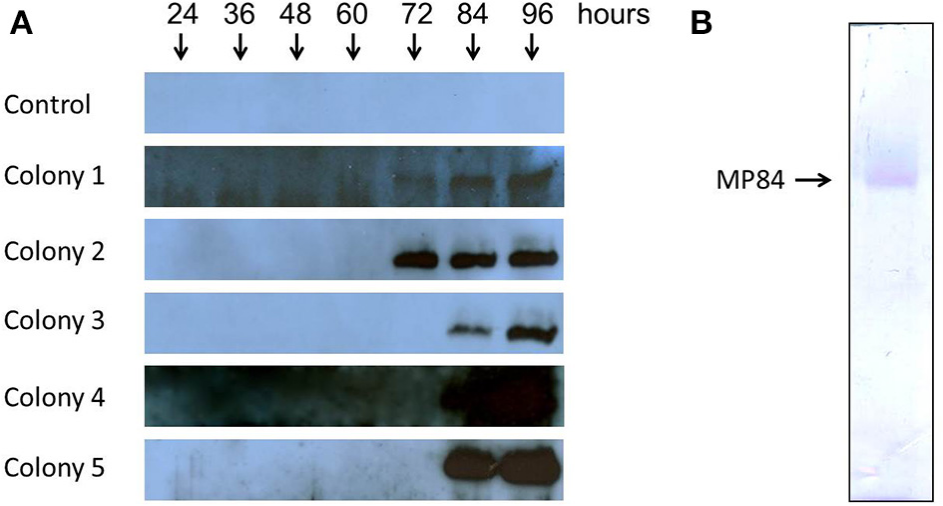
**Expression of MP84 mannoprotein in *P. pastoris***. *P. pastoris* was transformed with pPICZαB-MP84 plasmid and positive colonies were selected in the presence of the drug. Expression tests were performed to choose colonies with high levels of expression. Colony 2 was selected with a high amount of protein secreted. MP84 was then purified from the culture supernatant by affinity chromatography with nickel columns. **(A)** Western Blot analysis of the recombinant MP84 expression of the indicated colonies with 24, 36, 48, 60, 72, 84, and 96 hours of growth. A colony with pPICZαB without MP84 gene was used as negative control (Control). **(B)** SDS-PAGE of *P. pastoris* colony 2 culture supernatant after purification by affinity chromatography with nickel columns.

After the screening of transformants with zeocin antibiotic, expression tests were performed on five selected colonies, which verified the duration and expression levels of MP84 recombinant protein for each colony. The duration and expression levels were evaluated by Western Blot assays (Figure 3A), using anti-histidine as primary antibody. The five clones were grown in culture medium containing methanol for 24, 36, 48, 60, 72, 84, and 96 h. The Western Blot analyses demonstrated that all colonies tested were able to express and secrete recombinant MP84 to the culture medium. However, colony 2 was chosen for large-scale purification due to its capacity to secrete a large amount of integrated MP earlier (72 h) than the other clones (Figure 3A).

Large-scale purification of recombinant MP84 protein secreted from methanol-induced yeast cells was performed by concentration of cell-free growth medium, followed by affinity purification by an immobilized metal affinity chromatography (IMAC) using precharged Ni Sepharose™ High Performance (His Trap HP, Amersham Biosciences), as described in the Methods. The eluate was analyzed by SDS-PAGE, and the purity of the recombinant MP84 sample was attested (Figure 3B).

### Interaction of *C. neoformans* with A549 epithelial lung cells

**Figure 4:**
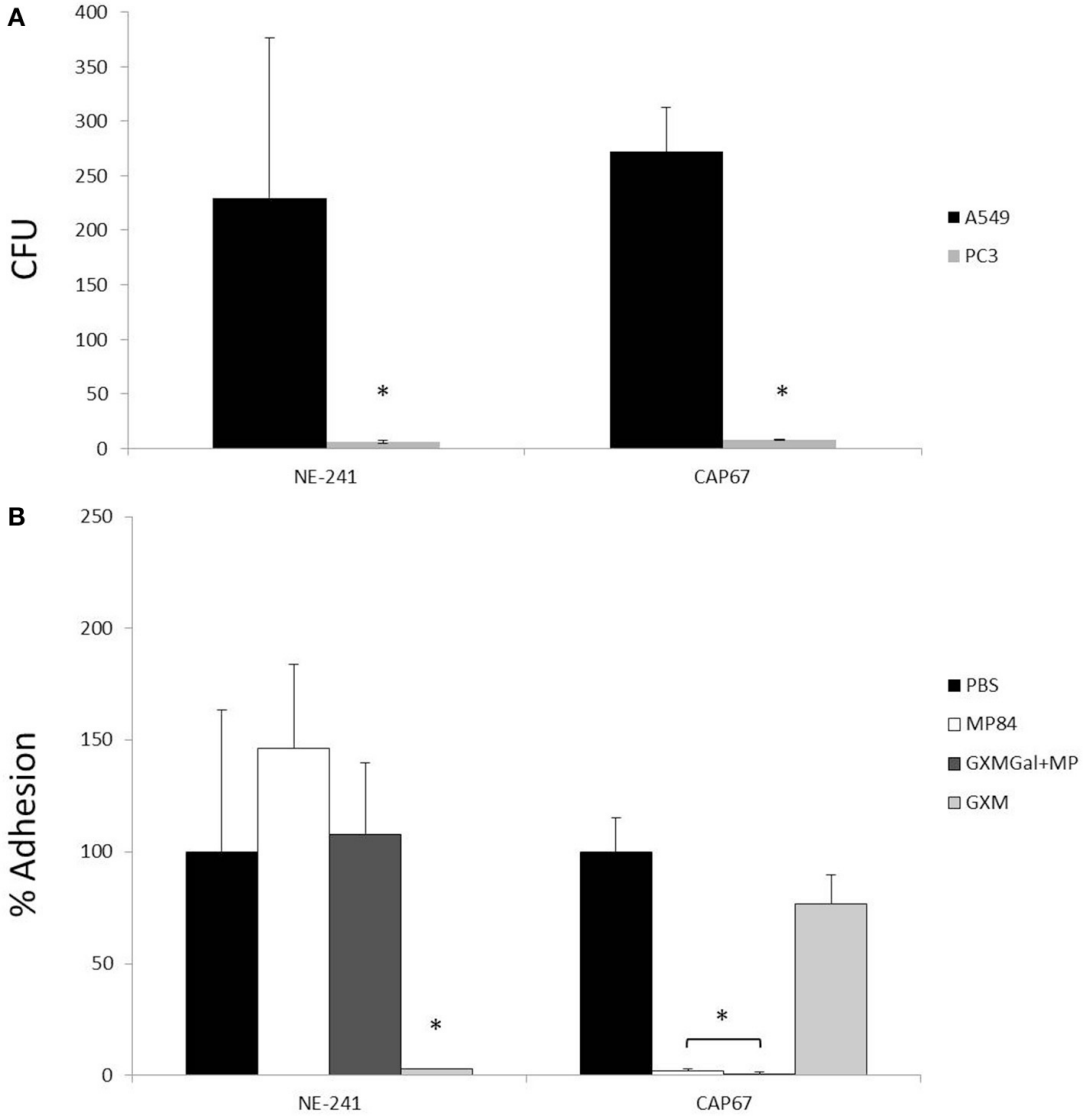
**Interaction of *C. neoformans* with epithelial lung cells**. Capsulated (NE-241) and non-capsulated (CAP67) *C. neoformans* strains were interacted with prostate human epithelial cells (PC3) or with epithelial lung cells (A549), with or without the presence of capsule constituents (GXM, GXMGal+MP or MP84). The interaction of *C. neoformans* with host cells was determined after counting colony forming units (CFUs) of viable yeasts recovered after 1 h of incubation. **(A)** CFUs of capsulated (NE-241) and non-capsulated (CAP67) strains incubated with A549 and PC3 cells. *C. neoformans* strains adhere significantly more to A549 than to PC3 cells. No significant difference was observed between the adhesion levels of the two strains. **(B)** Percentage of adhesion of *C. neoformans* strains to A549 cells in the presence or not of capsule constituents. Pretreatment of host cells with GXM inhibited NE-241 adhesion, while the incubation of lung cells with GXMGal+MP or MP84 inhibited the adhesion of CAP67. ^*^p < 0.05.

Adhesion is the first step to colonization and dissemination into the host. This observation leads us to study the binding between *C. neoformans* strains and host cells. Capsulated (NE-241) and non-capsulated (CAP67) strains were incubated for 1 h with epithelial lung cells or with prostate human epithelial cells (PC3). After culture and CFU counting, we observed that both strains adhered significantly to A549 cells, compared with PC3 cells (*p* < 0.05), used as a negative control (Figure 4A). We also observed that capsule size did not interfere in the interaction between the fungal cells and the epithelial lung cells, due to the capacity of both strains to bind to A549 cells. Additionally, no significant differences were observed in the adhesion levels between the two strains (*P* > 0.05) (Figure 4A). This result corroborates previous study, from Barbosa et al. (2006), which showed that *C. neoformans* yeast cells were able to specifically bind to A549 epithelial lung cells by capsular envelope components, such as GXM, however, disagrees with Merkel and Scofield (1997) who observed that the non-capsulated strain was the most adherent strain under the tested conditions.

Furthermore, we aimed to investigate the molecules involved in this interaction. To test the adhesion inhibition by capsule components, GXM, GXMGal+MP and recombinant MP84 were pre-incubated with A549 cells before the addition of fungal cells. We observed that GXM inhibits 85% of the adhesion of the capsulated strain to A549 cells, contrarily; this polysaccharide did not affect the interaction between the mutant strain and the host cells (Figure 4B). In addition, 90% of the adhesion of the non-capsulated strain (CAP67) was inhibited by GXMGal+MP fraction or recombinant MP84, which was not observed with the capsulated strain (NE-241) (Figure 4B).

### Binding of recombinant MP84 to A549 epithelial lung cells

The results from the experiments of interactions between fungal and host cells suggest that when GXM, GXMGal or MP84 putative receptors are blocked, *C. neoformans* cells become less adhesive to alveolar cells, leading us to investigate whether A549 cells are able to recognize MP84.

**Figure 5:**
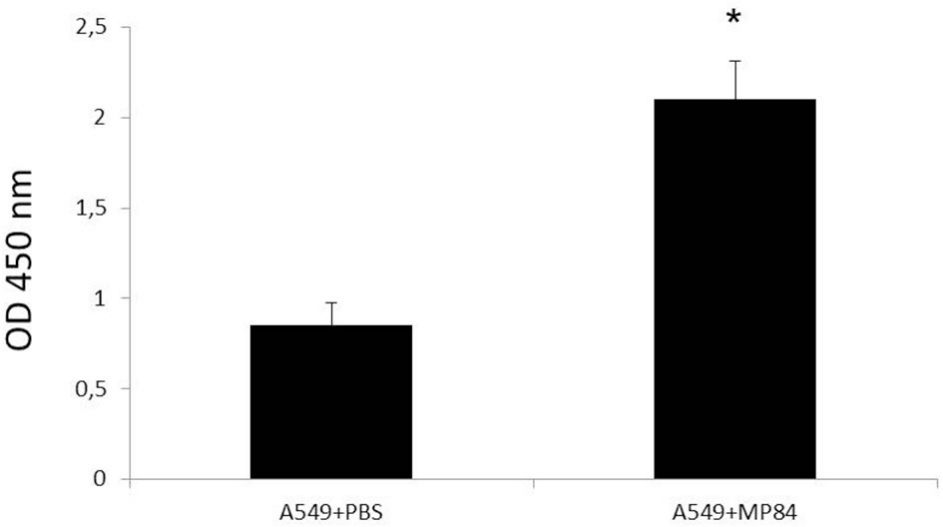
**Binding of recombinant MP84 to A549 cells was determinated by ELISA**. Incubation of epithelial lung cells with recombinant MP84 for 1 h, for further reaction with the anti-histidine antibody and a mouse anti-IgG HRP conjugated, shows that A549 cells are able to bind recombinant MP84. Control preparations were submitted to the same steps, but the recombinant MP84 was replaced by PBS. ^*^p < 0.05.

Epithelial lung cells, cultivated in 96-well plate, were incubated with purified recombinant MP84 and analyzed by ELISA with anti-histidine antibody, as demonstrated in Figure 5. After 1 h of interaction with recombinant MP84, the A549 cells were strongly recognized by the anti-histidine antibody, suggesting the existence of MP binding sites in the host cells.

## Discussion

*C. neoformans* infection begins in the lung, by the inhalation of desiccated basidiospores or yeasts living in the environment, mainly in pigeon guano. In some cases, this fungus can surpass the local host defense and reach the central nervous system, causing fungal meningoencephalitis (Rodrigues et al., 1999). This observation implies that for a successful infectious process, *C. neoformans* must interact with different host tissues, such as the lung epithelia, endothelial cells and the blood brain barrier (Goldman et al., 1994; Chen et al., 2003; Chang et al., 2004).

After inhalation, *C. neoformans* cells are deposited into the alveolar space, and interact with types 1 and 2 epithelial cells, macrophages and lung surfactant proteins. In this regard, the ability of *C. neoformans* to interact with alveolar macrophages (Tucker and Casadevall, 2002) and to bind surfactant proteins (Van de Wetering et al., 2004) has been well studied; however, the mechanisms involved in the fungal interaction with epithelial cells remain largely unknown. Few studies reported the ability of *C. neoformans* yeasts to interact with human epithelial lung cells. In 1997, Merkel and Scofield demonstrated that capsulated and non-capsulated strains were able to adhere to A549 cells. They also observed that simple carbohydrates, such as sucrose, inositol, *N*-acetylglucosamine and *N*-acetylgalactosamine, as well as a monoclonal antibody produced against whole cryptococcal cells, inhibited adherence of *C. neoformans* to host cells, suggesting a specific host-pathogen interaction mediated by yeast adhesins (Merkel and Scofield, 1997). Later, Barbosa and co-authors demonstrated that the interaction between capsulated or acapsular *C. neoformans* yeasts and alveolar epithelia was mediated by GXM, allowing the fungus to reach the intracellular environment and damage host cells (Barbosa et al., 2006). The inhibitory activity of mannose or MPs was not evaluated in both studies.

Here, we observed that capsule size did not interfere in the interaction between the *C. neoformans* and human A549 human cells, since no significant differences (*P* > 0.05) were observed in the adhesion levels between the strains NE-241 (capsulated) and CAP67 (acapsular) with epithelial cells (Figure 4A). This suggests that in the absence of GXM, *C. neoformans* can utilize other capsule components as adhesins to interact with host cells. As MPs are internal capsule molecules that become most exposed in the absence of GXM, we aimed to investigate whether MPs could function as *C. neoformans* adhesin and mediate the interaction between the fungus and the host cells. For this, the first step was to obtain a good volume of MPs. This was achieved by the obtainment of a GXMGal+MP fraction, purified from the culture supernatant, but mainly, by the clone and expression of MP84 in *P. pastoris* yeasts.

MP84 was first identified in 2005, by Biondo and co-workers, together with MP115. N-terminal amino acid sequences of both proteins were used to search the *C. neoformans* nucleotide databases, and homologous genomic sequences were used to synthesize DNA probes and isolate cDNA clones containing the full-length genes.

Analysis of the gene sequence showed the presence of a serine/threonine-rich region, a potential site for heavy N-glycosylation, which was confirmed by marked shifts in the molecular mass of MP84 (23 kDa) after peptide-*N*-glycosidase F treatment (Biondo et al., 2005). Moreover, homology with chitin deacetylases from other organisms was found during analysis of the deduced amino acids sequence. Chitin deacetylases are enzymes that convert chitin to chitosan, the deacetylated form of chitin. Chitosan is indispensable for fungal cell architecture, by maintaining cell integrity, normal capsule and bud separation (Levitz et al., 2001; Biondo et al., 2002). Additionally, MP84 contains a putative GPI anchor motif in the C-terminal portion. Since GPI anchors are used to link proteins to the cell wall or to the cell membrane, the presence of a putative GPI anchor site suggests a cell wall or capsule inner layer MP84 localization (Biondo et al., 2005).

In the present study, MP84 was, for the first time, expressed using the *P. pastoris* yeast model, with the aim to obtain a good amount of glycosylated recombinant protein, which is not possible using *E. coli* model. Mannosylation seems to be strongly required for MP functions. Unglycosylated MP, for example, had a greatly impaired capacity to stimulate antigen-specific IL-2 production from CD4^+^ T cells, compared with the mannosylated protein (Specht et al., 2007). In addition, the polysaccharide GXM was reported as mediator of host-pathogen interaction (Barbosa et al., 2006). These observations reinforced the necessity to use the *P. pastoris* model to express MP84. For this, two sense primers were constructed based on the MP84 gene sequence accessed in EMBL Nucleotide Sequence Database with AJ938050 accession number. These primers were used to amplify the MP84 gene from cDNA obtained from *C. neoformans* RNA (Figure 1). The MP84 amplified gene had to be bound to the expression vector (pPICZαB) and transformed into *E. coli* for large scale amplification of the insert-vector before being transformed into *P. pastoris*. Differential digestion assays confirmed the correct binding between the MP84 gene and pPICZαB (Figure 2), and the analysis of the insert-vector sequence confirmed that the insert was in frame with the α-factor signal sequence as well as with the C-terminal c-myc and HIS_6_ epitopes encoded by the vector (data not shown). Finally, the pPICZαB+MP84 gene was transformed into *P. pastoris* yeasts and expression tests were performed to verify the colonies expression levels and select the most secretory colony. Colony 2 was chosen to express the recombinant MP84 in large-scale, and this protein was purified by affinity chromatography in a nickel column (Figure 3).

Recombinant MP84, GXM or GXMGal+MP fraction was incubated with epithelial lung cells to test their capacity to inhibit the adhesion between fungal and host cells. Capsulated (NE-241) and non-capsulated (CAP67) *C. neoformans* strains were interacted with host cells. We observed that the fractions containing MP, as recombinant MP84 and GXMGal+MP, only inhibited the non-capsulated strain adhesion, whereas GXM only inhibited the capsulated strain adhesion (Figure 4B). These results led us verify the existence of putative binding sites that could recognize MPs on the surface of epithelial lung cells. Thus, we incubated recombinant MP84 with A549 cells, and after 1 h, we observed a specific interaction between this host cell and that MP (Figure 5).

Adhesion is one of the most important steps for the establishment of an infection. The outer layer molecules of microorganisms are the first antigens to interact with the host substrate, functioning as adhesins. *C. neoformans* yeasts are typically highly capsulated, and as GXM is abundant in this fungus capsule (more than 90%), this polysaccharide can mediate the interaction with host cells, as described elsewhere (Barbosa et al., 2006). However, not all *C. neoformans* wild yeasts are highly capsulated; this fungus exhibits striking variations in cellular structure and size, which have important consequences during infection. The morphological variations in *C. neoformans* can be divided into three classes, as reviewed by Zaragoza in 2011: (1) changes in capsule structure, (2) changes in capsule size, and (3) changes in the total size of the cell. These variations have profound consequences on the interaction with the host, involving survival, phagocytosis escape and immune evasion and dissemination.

Changes in cell size can be achieved by the formation of cryptococcal giant/titan cells or microforms. Giant/titan cells, which are most common during chronic infection, help *C. neoformans* evade the host immune system, since they avoid macrophage phagocytosis (Okagaki et al., 2010; Okagaki and Nielsen, 2012). In contrast, in recent review, Zaragoza hypothesized that micro cells could have a particular ability to disseminate and cross biological barriers, such as endothelia and the brain–blood barrier, and in consequence, contribute to the development of cryptococcal meningitis (Zaragoza, 2011). Changes in capsular structures mainly occur during the crossing of the brain–blood barrier, suggesting that capsular variations are required for dissemination and organ colonization (Garcia-Hermoso et al., 2004; Charlier et al., 2005). The last variation in cell morphology described by Zaragoza (2011) is the change in capsule size. It is well known that the capsule is an important *C. neoformans* virulence factor, indispensable for the establishment of cryptococcosis, however, in the environment, before inhalation, the fungus capsule is still small, but significantly increases in size after a few hours of post-infection (Feldmesser et al., 2001). This observation led us to hypothesize that in the first moments of the interaction between *C. neoformans* and alveolar epithelia, just after the inhalation of poorly encapsulated yeasts, the fungus can utilize other molecules besides GXM to adhere to host cells, due to the low amount of this polysaccharide in the thin capsule. Since there are big variations in morphology of *C. neoformans* cells, both highly capsulated and poorly capsulated cells have the ability to interact with host cells during infection. Here, we demonstrated, for the first time, the involvement of MPs in the interaction of *C. neoformans* to epithelial lung cells.

### Conflict of interest statement

The Guest Associate Editor Eliana Barreto-Bergter declares that, despite being affiliated to the same institution as authors Teixeira, Penha, Mendonça-Previato and Previato, the review process was handled objectively and no conflict of interest exists. The authors declare that the research was conducted in the absence of any commercial or financial relationships that could be construed as a potential conflict of interest.
